# Scaffold-fused riboregulators for enhanced gene activation in *Synechocystis* sp. PCC 6803

**DOI:** 10.1002/mbo3.257

**Published:** 2015-04-10

**Authors:** Yuta Sakai, Koichi Abe, Saki Nakashima, James J Ellinger, Stefano Ferri, Koji Sode, Kazunori Ikebukuro

**Affiliations:** 1 Department of Biotechnology and Life Science, Graduate School of Engineering, Tokyo University of Agriculture and Technology2-24-16 Naka-cho, Koganei, Tokyo, 184-8588, Japan; 2 CREST, Japan Science and Technology Agency2-24-16 Naka-cho, Koganei, Tokyo, 184-8588, Japan

**Keywords:** Hfq, posttranscriptional gene regulation, riboregulator, *Synechocystis* sp. PCC 6803, synthetic biology

## Abstract

Cyanobacteria are an attractive host for biofuel production because they can produce valuable chemical compounds from CO_2_ fixed by photosynthesis. However, the available genetic tools that enable precise gene regulation for the applications of synthetic biology are insufficient. Previously, we engineered an RNA-based posttranscriptional regulator, termed riboregulator, for the control of target gene expression in cyanobacterium *Synechocystis* sp. PCC 6803. Moreover, we enhanced the gene regulation ability of the riboregulators in *Escherichia coli* by fusing and engineering a scaffold sequence derived from naturally occurring *E. coli* noncoding small RNAs. Here, we demonstrated that the scaffold sequence fused to the riboregulators improved their gene regulation ability in *Synechocystis* sp. PCC 6803. To further improve gene regulation, we expressed an exogenous RNA chaperone protein that is responsible for noncoding small RNA-mediated gene regulation, which resulted in higher target gene expression. The scaffold sequence derived from natural *E. coli* noncoding small RNAs is effective for designing RNA-based genetic tools and scaffold-fused riboregulators are a strong RNA-tool to regulate gene expression in cyanobacteria.

## Introduction

Cyanobacteria have great potential as a host for biofuel production owing to their photosynthesis ability, higher growth rate than plants, and the ease of genetic engineering. They fix CO_2_ from air by photosynthesis and convert it into chemical products via biosynthetic pathways (Niederholtmeyer et al. 2010; Oliver et al. 2013; Osanai et al. 2013). It is important to improve the productivity of valuable compounds. Recent reports demonstrated that optimizing the expression level of genes involved in biosynthetic pathways to balance the metabolic flux by using genetic tools enables higher yields in several organism, such as *Escherichia coli* (Zhang et al. 2012; Na et al. 2013; Xu et al. 2013). However, while diverse and powerful gene regulators have been designed for *E. coli*, the genetic tools engineered in cyanobacteria are limited to only a few inducible promoters (Huang et al. 2010; Huang and Lindblad 2013; Abe et al. 2014a; Camsund et al. 2014) or RNA-based tools (Nakahira et al. 2013; Abe et al. 2014b). To expand the synthetic biology applications using cyanobacteria, more diverse and powerful gene regulators are required.

**Figure 1:**
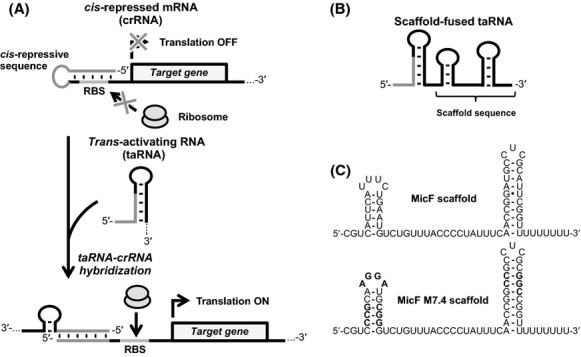
The mechanism of riboregulator for target gene regulation and the scaffold-fused taRNA. (A) Expression of a target gene located downstream of the ribosome-binding site (RBS) of crRNA is induced in presence of taRNA, which exposes the RBS upon taRNA-crRNA hybridization. (B) Scaffold-fused taRNA harbors a sRNA scaffold sequence at the 3′-end. (C) Predicted secondary structure of sRNA scaffold sequences used in this study. The MicF scaffold is derived from natural *Escherichia coli* MicF sRNA. MicF M7.4 scaffold harbors GC-rich stem structures and a mutated loop region (noted in bold letters).

RNA-based gene regulators allow fine-tuning of the target gene and are easy to design because the regulation mechanism is mediated by base pairing against the target mRNA sequence. By focusing on these advantages, we previously engineered a riboregulator, an RNA-based genetic tool that activates gene expression posttranscriptionally, to control the expression of a target gene in cyanobacteria (Abe et al. 2014b). A riboregulator is composed of two RNA parts: a *cis*-repressed mRNA (crRNA) and a noncoding *trans*-activating RNA (taRNA) (Fig.1A) (Isaacs et al. 2004). The crRNA harbors a short sequence in its 5′-untranslated region (UTR) that hybridizes the ribosome-binding site (RBS) and forms a stem loop structure to block the RBS from the ribosome binding, resulting in the target gene repression. The taRNA contains a complementary sequence to the crRNA and can activate the target gene expression. The hybridization between taRNA and crRNA unwinds the stem loop structure of crRNA and exposes the RBS to induce the translation of the target gene. To engineer a riboregulator suitable for cyanobacteria, we first designed a crRNA (crR*2) that incorporates a strong RBS sequence (RBS*) in cyanobacterium *Synechocystis* sp. PCC 6803 (*Synechocystis*) (Heidorn et al. 2011), and then designed a cognate taRNA, taR*2 (Abe et al. 2014b). We demonstrated the designed riboregulator, taR*2 and crR*2 was able to regulate target gene expression in *Synechocystis*.

In addition, we reported that the gene regulation ability of taRNAs could be improved by fusing to its 3′-end a natural noncoding small RNA (sRNA)-derived scaffold sequence in *E. coli* (Fig.1B) (Sakai et al. 2014). The sRNA scaffold includes the Hfq-binding and rho-independent transcription terminator sequences. Hfq is an RNA chaperone protein that plays an important role in *trans*-encoded sRNAs-mediated gene regulation (Vogel and Luisi 2011) and is conserved in a wide-range of bacteria including *Synechocystis* (Dienst et al. 2008). In particular, *E. coli*-derived Hfq has been well studied and the Hfq binds to the single-stranded AU-rich sequence within the scaffold region that is located outside of the antisense region. This Hfq-binding enhances the stability of sRNAs in vivo by protecting them from nuclease cleavage and can also promote the hybridization against the target mRNA (Møller et al. 2002; Zhang et al. 2002).

In the present study, we aimed to improve the gene regulation ability of our riboregulator in *Synechocystis* for better control of target gene expression. The scaffold-fused taR*2 constructs that enhanced the target gene expression in *E. coli* was tested in *Synechocystis* and their function were compared with taR*2 that we previously reported (Abe et al. 2014b). The scaffold-fused taRNAs exhibited higher gene regulation abilities in *Synechocystis* cells and were further examined in *Synechocystis* expressing *E. coli*-derived Hfq.

## Material and Methods

### Materials

All oligonucleotides used in this research were obtained from Operon Biotechnologies, Inc. (Huntsville, AL) and are listed in Table S1. All BioBrick standard biological parts were obtained from the Registry of Standard Biological Parts (http://partsregistry.org).

### Plasmids and bacterial strains

The plasmids used in this research were constructed based on previously constructed plasmids (Abe et al. 2014b; Sakai et al. 2014) using standard cloning techniques and are listed in Table1. All constructs were subcloned into the pKTNEP vector, which is the broad-host-range pKT230 plasmid backbone (Bagdasarian et al. 1981) with a cloning site that is compatible with BioBrick standards (Shetty et al. 2008). Plasmids were constructed using *E. coli* DH5*α*. The constructed plasmids were transformed into *Synechocystis* sp. PCC 6803 as described previously (Abe et al. 2014b). The transformed *Synechocystis* sp. PCC 6803 cells were cultured in BG-11 medium containing appropriate antibiotics at 30°C.

**Table 1:** Plasmids used to evaluate the riboregulator in *Synechocystis* sp. PCC 6803

Abbreviation	Full construct name
Empty vector	pKTNEP (no insert)
taR^*^2/crR^*^2	pKTNEP-P_*nrsB*_-taR^*^2-DT-P_*trcΔlacO*_-crR^*^2-GFPuv-DT
taR^*^2-MicF/crR^*^2	pKTNEP-P_*nrsB*_-taR^*^2-MicF-DT-P_*trcΔlacO*_-crR^*^2-GFPuv-DT
taR^*^2-MicF M7.4/crR^*^2	pKTNEP-P_*nrsB*_-taR^*^2-MicF M7.4-DT-P_*trcΔlacO*_-crR^*^2-GFPuv-DT

DT stands for double terminator (BioBrick™ BBa_B0015).

### GFPuv assay

Transformants of *Synechocystis* sp. PCC 6803 were cultured and evaluated as described previously (Abe et al. 2014b). Briefly, transformants were cultured in 50 mL BG-11 medium containing appropriate antibiotics until optical density at 730 nm reached to approximately 0.5. Then f.c. 20 *μ*mol/L NiSO_4_ was added into the culture to induce taR*2’s transcription. After 15 h of incubation, cells from 1 mL of the culture were harvested by centrifugation (8000*g*, 5 min), washed by resuspending in 240 *μ*L of BG-11 medium, recentrifuged, and resuspended in 240 *μ*L of BG-11 medium. 200 *μ*L was transferred to a 96-well microtiter plate and GFPuv fluorescence and optical density at 730 nm were measured using a micro plate reader Varioskan Flash (Ex: 395 nm, Em: 509 nm; Thermo Fisher, Rockford, IL). The GFPuv fluorescence was normalized with the values of cell growth (OD_730_) to calculate cellular fluorescence. The background cellular fluorescence of *Synechocystis* sp. PCC 6803 transformed with pKTNEP (empty vector) was subtracted from each sample.

### RNA preparation and northern blot analysis

Transformants of *Synechocystis* sp. PCC 6803 were treated with 300 *μ*g/mL of rifampicin and total RNAs were purified using Tri-Reagent (Molecular Research Center, Inc., Cincinnati, OH) according to the manufacturer’s instructions. The purified total RNA was subjected to northern blot analysis using 5′-biotinylated DNA probes (Table S1) as described previously (Sakai et al. 2014).

### Western blot analysis

The soluble fraction of *Synechocystis* sp. PCC 6803 *Ecohfq*::*kan* cells was prepared by collecting the supernatant after centrifuging (15,000*g*, 15 min, 4°C) the fractured cells. The soluble fraction was separated by sodiumdodecyl sulphate polyacrylamide gel electrophoresis (SDS-PAGE) and transferred to a nitrocellulose membrane. The membrane was blocked by incubation with TBS-T buffer (10 mmol/L Tris, pH 7.4, 150 mmol/L NaCl, 5 mmol/L KCl, 0.5% Tween 20) containing 10% skim milk and then incubated with anti-His_6_-tag antibody (Medical & Biological Laboratories, Nagoya, Japan) for 1 h. The membrane was washed three times with TBS-T buffer and incubated with a 1:5000 dilution of horseradish peroxidase-conjugated anti-mouse IgG antibody (Promega, Madison, WI) for 1 h. After washing the membrane three times with TBS-T buffer, the His_6_-tag-fused Hfq was visualized with Immobilon western chemiluminescent HRP substrate (Millipore, Billerica, MA), followed by the detection using the ImageQuant LAS 4000 mini system (GE Healthcare, Munich, Germany).

## Result and Discussion

**Figure 2:**
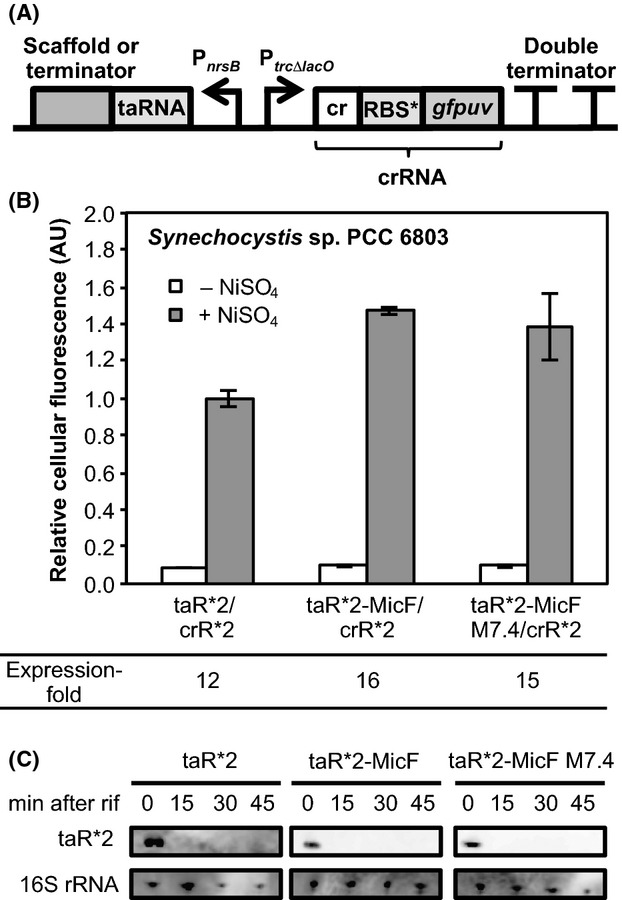
Scaffold-fused taR*2’s in *Synechocystis* sp. PCC 6803. (A) Schematic representation of the construct to evaluate the taR*2’s in *Synechocystis* sp. PCC 6803. (B) The taR*2’s were evaluated in *Synechocystis* sp. PCC 6803. The transcription of taR*2’s was induced in Ni^2+^-inducible *nrsB* promoter. The cellular fluorescence of taR*2 in the presence of NiSO_4_ was normalized to 1.0. The expression-fold representing the ratio of GFPuv expression levels in the presence and absence of NiSO_4_ are shown. The graphs depict the mean and error bars represent the standard deviation of experiments performed in triplicate. (C) Northern blot analysis of taR*2’s. The transcription was stopped by adding rifampicin and cells were harvested at the indicated time points for RNA preparation. Total RNA was analyzed using probes specific for taR*2s and 16S rRNA, respectively.

Two scaffold-fused taRNAs, taR*2-MicF and taR*2-MicF M7.4 were evaluated in *Synechocystis*. This MicF M7.4 scaffold was engineered by substituting the AU and GU-base pairs present in the stem loop structures of MicF scaffold into GC-base pairs and also by replacing the loop region with an Hfq high-affinity sequence (Link et al. 2009). This engineered MicF scaffold was predicted to form a stable secondary structure from M-fold secondary structure prediction (Zuker 2003). In our previous study, a naturally occurring *E. coli* MicF sRNA-derived scaffold sequence was revealed to be a suitable scaffold sequence to improve the gene regulation abilities of taR*2 (Fig.1C) (Sakai et al. 2014). While the taR*2-MicF harboring an intact MicF sRNA-derived scaffold sequence enhanced the gene expression to 2.5-fold in *E. coli*, taR*2-MicF M7.4 harboring the engineered MicF scaffold further enhanced the gene expression to 1.5-fold, which was 4.1-fold higher than taR*2 without the scaffold sequence. Moreover, the scaffold-fused taRNAs were more stable than the taRNA without the scaffold sequence in vivo and had low function in *E. coli*Δ*hfq* strain, indicating that the endogenous Hfq bound and protected the scaffold-fused taRNAs from nuclease cleavage as well as natural *trans*-encoded sRNAs. To evaluate taR*2-MicF and taR*2-MicF M7.4 in *Synechocystis*, the scaffold-fused taR*2s were inserted downstream of the Ni^2+^-inducible *nrsB* promoter (P_*nrsB*_) (Lopez-Maury et al. 2002) and the crR*2 was constitutively transcribed from the *trc* promoter without the *lac* operator sequence (P_*trcΔlacO*_) (Fig.2A). The *gfpuv* gene was inserted under crR*2 as a reporter gene to construct plasmids to evaluate the riboregulators (Table1).

In the presence of NiSO_4_, the taR*2-MicF and taR*2-MicF M7.4 increased the GFPuv expression 1.4-fold more than taR*2 alone, demonstrating that the fusion of a natural *E. coli* sRNA-derived scaffold was also effective to improve the gene regulation abilities of taR*2’s in *Synechocystis* (Fig.2B). Unlike our observations that taR*2-MicF M7.4 activated gene expression 1.5-fold higher than taR*2-MicF in *E. coli*, both taR*2-MicF and taR*2-MicF M7.4 showed similar gene regulation abilities in *Synechocystis*. However, we note that fold expression of GFPuv under NiSO_4_ induction relative to uninduced samples was approximately 16-fold and 15-fold for taR*2-MicF and taR*2-MicF M7.4, respectively, which were higher than taR*2. These improvement might be due to fusing MicF or MicF M7.4 scaffold affected the secondary structure of taR*2 so that can have higher hybridization efficiency against crR*2. These results show that scaffold-fused taR*2 might be a powerful genetic tool for controlling gene expression in *Synechocystis*.

To investigate whether the endogenous Hfq in *Synechocystis* was responsible for the high gene regulation ability of scaffold-fused taR*2s, we performed northern blot analysis to evaluate the stabilities of taR*2’s. While our previous data showed that taR*2-MicF and taR*2-MicF M7.4 were more stable than taR*2 in *E. coli* cells, they were all unstable and had short half-lives in *Synechocystis* (Fig.2C). This could be due to the difference in the preferable binding sequence between *Synechocystis*-derived Hfq and *E. coli*-derived Hfq. Although the binding between MicF sRNA *Synechocystis*-derived Hfq has not been investigated to date, *Synechocystis*-derived Hfq has low binding affinity against natural *E. coli* sRNA in vitro and does not enhance the stabilities of *E. coli* sRNA when *Synechocystis*-derived Hfq was expressed in *E. coli* Δ*hfq* cells (Bøggild et al. 2009). Therefore, the fusion of MicF scaffold might not enhance the stability of taR*2s in *Synechocystis* because taR*2-MicF and taR*2-MicF M7.4 could not be protected from nuclease degradation due to the non or weak binding affinity of endogenous Hfq in vivo. Alternatively, the fusion of the MicF or the engineered MicF M7.4 scaffold might affect the stabilities of secondary structure of taR*2 for higher hybridization efficiency against crR*2 to achieve higher gene expression when the taR*2 transcription was induced.

**Figure 3:**
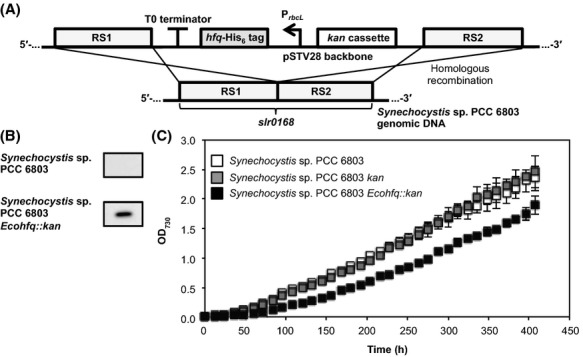
*Synechocystis* sp. PCC 6803 expressing *Escherichia coli*-derived Hfq. (A) Schematic representation of the integration of *E. coli*-derived Hfq via homologous recombination. His_6_-tag-fused *hfq* was regulated by *rbcL* promoter and integrated into the neutral site of *Synechocystis* sp. PCC 6803 genomic DNA. (B) The expression of His_6_-tag-fused Hfq in *Synechocystis* sp. PCC 6803 *Ecohfq::kan* cells was confirmed by western blot analysis using anti-His_6_-tag antibody. (C) The growth curve analysis of *Synechocystis* sp. PCC 6803 cells integrated with the *kan* cassette (*Synechocystis* sp. PCC 6803 *kan*), and *Synechocystis* sp. PCC 6803 *Ecohfq::kan*. The graphs depict the mean and error bars represent the standard deviation of experiments performed in triplicate.

To further improve the gene regulation ability of scaffold-fused taR*2, we aimed to utilize the Hfq protein in *Synechocystis*. In contrast to *E. coli*-derived Hfq, the Hfq-dependent natural sRNAs or the preferable binding sequence of *Synechocystis*-derived Hfq has not been well investigated. Therefore, we chose to introduce and express *E. coli*-derived Hfq in *Synechocystis*. Since the scaffold-fused taRNAs had stronger function and were more stable than the taRNAs without the scaffold sequence in *E. coli*, likely due to the Hfq binding, the scaffold-fused taR*2 might show improved gene regulation ability in *Synechocystis* expressing *E. coli*-derived Hfq. Therefore, we integrated *E. coli*-derived Hfq into the genomic DNA of *Synechocystis* via homologous recombination. The *hfq* gene was cloned from *E. coli* K-12 genomic DNA and a his_6_-tag was fused to the C-terminus and the expression was regulated by the *rbcL* promoter (P_*rbcL*_). This *E. coli*-derived Hfq expressing cassette was inserted into the integration vector, pSTVISK, which enables integration of the target DNA sequence and a kanamycin cassette into *slr0168*, a well-known neutral site (Fig.3A) (Williams 1988). The *Synechocystis* strain into which *E. coli*-derived Hfq was integrated, termed *Synechocystis Ecohfq*::*kan*, was selected on BG-11 agar plates containing kanamycin at a final concentration of 20 *μ*g/mL. Expression of *E. coli*-derived Hfq was confirmed by western blot analysis using anti-His_6_-tag antibody (Fig.3B). However, the expression of *E. coli*-derived Hfq reduced the growth rate slightly (Fig.3C). This could be due to the variation in the level of endogenous RNA caused by the binding of *E. coli*-derived Hfq. It has been shown that the binding affinity of *E. coli*-derived Hfq against their preferable RNA sequence is strong (*K*_D_ ∼ 10^−8^ mol/L) (Link et al. 2009), therefore binding against *Synechocystis* endogenous RNA could occur. Although, this slower growth rate might be improved by optimizing the expression level of *E. coli*-derived Hfq.

**Figure 4:**
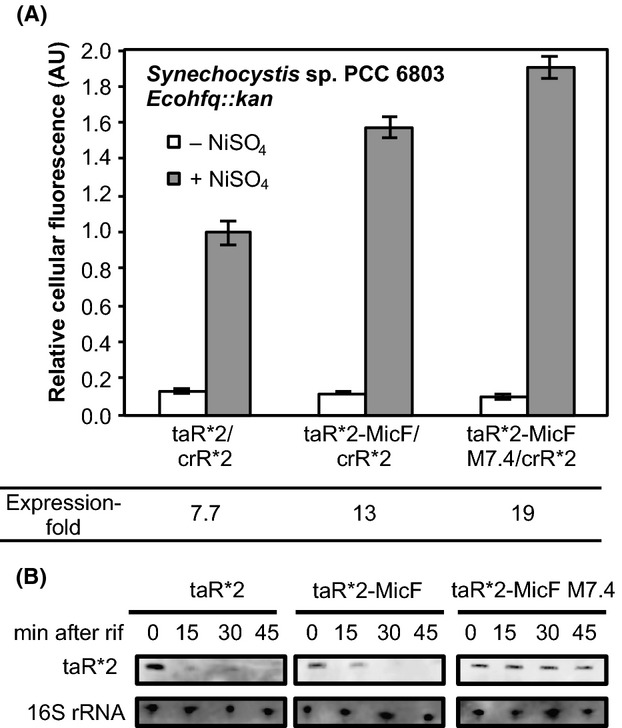
Scaffold-fused taR*2s in *Synechocystis* sp. PCC 6803 *Ecohfq::kan*. (A) The taR*2’s were evaluated in *Synechocystis* sp. PCC 6803. The transcription of taR*2’s were induced Ni^2+^-inducible *nrsB* promoter. The cellular fluorescence of taR*2 in the presence of NiSO_4_ was normalized to 1.0. The expression-fold representing the ratio of GFPuv expression levels in the presence and absence of NiSO_4_ are shown. The graphs depict the mean and error bars represent the standard deviation of experiments performed in triplicate. (B) Northern blot analysis of taR*2’s. The transcription was stopped by adding rifampicin and cells were harvested at the indicated time points for RNA preparation. Total RNA was analyzed using probes specific for taR*2 and 16S rRNA, respectively.

The three taRNAs, taR*2, taR*2-MicF, and taR*2-MicF M7.4 were introduced into *Synechocystis Ecohfq*::*kan* to evaluate the riboregulators and investigate whether the gene regulation ability could be enhanced in cyanobacteria strains expressing *E. coli*-derived Hfq. We transformed *Synechocystis Ecohfq*::*kan* cells using the identical plasmids used to transform *Synechocystis* wild-type cells and cultured the cells as described previously (Abe et al. 2014b) except kanamycin (f.c. 20 *μ*g/mL) was added to the medium. As a result, only taR*2-MicF M7.4 enhanced its gene regulation ability in *Synechocystis Ecohfq*::*kan* (Fig.4A). The taR*2-MicF M7.4 exhibited the highest fold expression and was approximately 1.5-fold higher than taR*2-MicF and 2.5-fold higher than taR*2. This result demonstrates that the engineered scaffold sequence was effective toward improving the function of sRNA in *Synechocystis*. Meanwhile, the fold expression of taR*2 and taR*2-MicF decreased when the *E. coli*-derived Hfq was expressed. This might be due to the effect of the slower growth rate. The stabilities of the taR*2s were evaluated by northern blot analysis and revealed that taR*2-MicF M7.4 was more stable than taR*2 and taR*2-MicF (Fig.4B). Meanwhile, taR*2-MicF was slightly more stable than taR*2. These observation suggests that the *E. coli*-derived Hfq binds to both taR*2-MicF and taR*2-MicF M7.4 in *Synechocystis Ecohfq*::*kan*, although the binding against taR*2-MicF M7.4 might be stronger. This different binding affinity might be due to the stability of secondary structures of the two scaffold sequences. The taR*2-MicF M7.4 harbors an engineered MicF scaffold, in which the mutations were introduced into the two stem-loop structures and was predicted to form a stable secondary structure. The single-stranded AU-rich region of the MicF scaffold is the predicted Hfq-binding site. This region may be more exposed in vivo for taR*2-MicF M7.4 than the nonengineered version, which would lead to increased *E. coli*-derived Hfq binding and consequently an increase of target gene translation. Although the stability of taR*2-MicF M7.4 was apparently higher than that of taR*2 and taR*2-MicF, the improvement in expression was lower than that observed in our previous study when taR*2-MicF M7.4 was tested in *E. coli* cells (Sakai et al. 2014). This observation may be due to the unspecific binding of *E. coli*-derived Hfq against the outside of the MicF M7.4 scaffold, such as the taR*2 region, which may have affected its hybridization with crR*2 to induce GFPuv translation.

To date, a variety of artificial noncoding sRNAs have been designed and applied to regulate metabolic pathways or toxic genes (Callura et al. 2010; Na et al. 2013). The majority of artificial sRNAs have been engineered in *E. coli* and utilization of scaffold sequences including the Hfq-binding region derived from naturally occurring sRNAs, has been demonstrated to be a valuable strategy for engineering highly functional Hfq-dependent RNA-based artificial gene regulators (Man et al. 2011; Sharma et al. 2012, 2013; Na et al. 2013; Sakai et al. 2014). Because of the lack of knowledge regarding the Hfq protein and Hfq-dependent sRNAs in bacteria except *E. coli*, and the time-consuming nature of this process, introducing *E. coli*-derived Hfq in other bacteria can be an effective strategy for utilizing RNA-based gene regulators engineered in *E. coli*. We demonstrated that artificial sRNAs can be an effective tool to regulate gene expression in cyanobacteria. Furthermore, a sRNA scaffold sequence derived from natural *E. coli* sRNA was useful as a component to design sRNAs in cyanobacteria since MicF scaffold fused to taR*2 was capable of improving the gene regulation ability, even when the *E. coli*-derived Hfq was not expressed.

## Conclusion

We tested the previously designed scaffold-fused taR*2 in *Synechocystis* and improved the gene regulation ability by utilizing *E. coli*-derived Hfq in *Synechocystis*. The scaffold-fused taR*2’s were composed of taR*2, a previously engineered riboregulator suitable for gene regulation in *Synechocystis*, and the sRNA scaffold sequence derived from natural *E. coli* MicF sRNA. The fusion of MicF and engineered MicF M7.4 scaffold sequence was effective to improve the gene regulation ability of taR*2 in *Synechocystis*. Moreover, taR*2-MicF M7.4 exhibited an increase in target gene activation in *Synechocystis* expressing *E. coli*-derived Hfq. This is the first report to demonstrate the expression of *E. coli*-derived Hfq in *Synechocystis* and to show that RNA-based gene regulators can be used in this engineered *Synechocystis* strain. Our results suggest that our scaffold-fused taR*2 can be a strong RNA tool to regulate gene expression in *Synechocystis* and to control genes for biofuel production.

## Supporting Information

**Table S1.** Oligonucleotides used in this research.
