# Supplementary material for: Scaffold-fused riboregulators for enhanced gene activation in Synechocystis sp. PCC 6803
Source: Microbiologyopen. 2015 Apr 10;4(4):533–40. doi: 10.1002/mbo3.257 (PMC4554450; doi:10.1002/mbo3.257)
Supplement: Supplementary file 1 [file mbo30004-0533-sd1.docx]

Supporting Information

Table S1. Oligonucleotides used in this research.

| ID | Sequence (5'→3') |
| --- | --- |
| pKTNEP-Fw | TTGCTGCAGTTCTGACAACGATGAGCTTGC |
| pKTNEP-Rev | CGAGAATTCGCTAGCTTACGAGCAGAAGAGCATACATCTGGAA |
| taR*2-probe | [biotin]-GATATATGGTAGTAGTAAGTTAATTTTCATTAACCACCACTACTACCTCAACTATTGATTTGGGT |
| 16SrRNA-probe | [biotin]-AAGTCATCATGCCCCTTACGCCTTGGGCTACACACGTACTACAATGGTCGGGACAACGGGCAGCGAGCTCGCGAGAGTAAGCGAATCCCAT |
| pKTFw | TTTCCTGGCTTTGCTTCCAGAT |
| pKTRev  VF2  VR  NotI-RS1-Fw  NdeI-RS2-Rev  NdeI-pSTV-Fw  NotI-pSTV-Rev  EcoRI-aphII-Fw  EPX-aphII-Rev  PrbcL-BBprefix-Fw  PrbcL-Hfq-Rev  PrbcL-Hfq-Fw  HfqHis-HindIII-Rev  HindIII-TT0-Fw  TT0-BBsurfix-Rev | TGACAGGCAACCAGTCAGAA  GTGCCACCTGACGTCTAAGAA  ATTACCGCCTTTGAGTGAGC  ATTTCTGCGGCCGCTGGATCCTGGGCTTCGGCTATGGTG  GGAACATATGGGATCCATCAAAAAAGGCTTCC  ATCCCATATGTGAGCGGCCTCATTTCTTATT  ATCCAGCGGCCGCCTGATTAATAAGATGATCTTCTTGAGATC  TGGGAATTCCTTGGTCTGACAGCTCGAGT  CGGGAATTCCCTGCAGATACTACTCTAGATTTATTCAACAAAGC  TGGAATTCGCGGCCGCATCTAGACAGTCAATGGAGAGCATTGCC  CATCTAGGTCAGTCCTCCATAAACATTGAATAGCCTAGCTTTCT  TTATGGAGGACTGACCTAGATGGCTAAGGGGCAATCTTTACAAG  GAGCAAGCTTAGTGGTGGTGGTGGTGGTGTTCGGTTTCTTCGCT  GAATAAGCTTGGACTCCTGTTGATAGATCCAG  CGTCTGCAGCGGCCGCTACTAGTGCTTGGATTCTCACCAATAAA |

All oligonucleotides were purchased from Operon Biotechnologies, Inc. (Huntsville, AL, USA).
